# Efficacy of Alpha-lipoic Acid in The Management of Diabetes Mellitus: A Systematic Review and Meta-analysis

**DOI:** 10.22037/ijpr.2019.1100842

**Published:** 2019

**Authors:** Mahmoud Ahmed Ebada, Notila Fayed, Laila Fayed, Souad Alkanj, Ahmed Abdelkarim, Haya Farwati, Aya Hanafy, Ahmed Negida, Mohamed Ebada, Yousef Noser

**Affiliations:** a *Faculty of Medicine, Zagazig University, Zagazig, El-Sharkia, Egypt. *; b *Al-Ahrar Teaching Hospital, Zagazig, El-Sharkia, Egypt. *; c *Department of Pathology, Faculty of Medicine, Zagazig University, Zagazig, El-Sharkia, Egypt.*

**Keywords:** Alpha-lipoic acid, Diabetes Mellitus, Thioctic Acid, Metabolic Diseases, Meta-analysis

## Abstract

Alpha-lipoic acid (ALA) is a naturally-occurring compound that has shown promising antioxidant and anti-inflammatory effects in experimental and human studies. The aim of this study was to assess the efficacy of ALA in the management of patients with diabetes mellitus (DM). We searched Medline (via PubMed), EBSCO, Scopus, and Web of Science for relevant randomized controlled trials. Data on glycated hemoglobin (HbA1c), blood glucose levels, lipid profile components, HOMA, and glutathione peroxidase (GPx) were extracted and pooled as the standardized mean difference (SMD) in a random effect model meta-analysis using RevMan version 5.3. Ten studies (n = 553 patients) were included. In the term of HBA1C, the overall SMD did not favor either of the two groups (SMD = 0.01, 95% CI [-0.32,0.35]; *p *= 0.94) in uncomplicated T2DM patients. Moreover, there was no statistically significant difference between the two groups in terms of FBG (SMD = -0.06, 95% CI [-0.44,0.33]; *p *= 0.78), PPBG (SMD = 0.04, 95% CI [-0.27,0.34]; *p *= 0.82), HDL (SMD = -0.05, 95% CI [-0.35,0.25]; *p *= 0.75), LDL (SMD = -0.05, 95% CI [-0.33,0.23]; *p *= 0.75). In terms of GPx, ALA was superior to placebo (SMD = 0.43, 95% CI [0.07,0.8]; *p *= 0.02). Our analysis showed that ALA was not superior to placebo in terms of HBA1C, LDL, HDL, TC, TG reduction in uncomplicated T2DM. However, in terms of GPx, ALA was significantly superior to the placebo. Further studies with larger sample sizes should investigate different doses of ALA in DM patients.

## Introduction

Diabetes Mellitus (DM) is a metabolic disorder characterized by high levels of blood glucose. Based on the capacity of insulin secretion from the pancreatic beta cells, it is classified into insulin-dependent type 1 DM with deficient insulin secretion (T1DM) and non-insulin-dependent type 2 DM with insufficient insulin sensitivity and glucose uptake in peripheral tissue (T2DM) (1, 2). 

Most of the T2DM patients suffer from obesity and lipoprotein metabolism disorders (3). Dyslipoproteinemia in diabetic patients is associated with increased levels of total cholesterol (TC), low-density lipoproteins (LDL) and triglycerides (TG), and diminished level of high-density lipoproteins (HDL) (3–5). 

Both obesity and Lipid metabolic changes are substantial risk factors for developing complications, especially microvascular ones (3, 6). Visceral fat is linked to elevated inflammatory mediators, subclinical inflammation, and diabetic polyneuropathy and it has been linked to oxidative stress, which is believed to play a role in insulin resistance and T2DM progression (7). 

Therefore, it is necessary to properly correct lipoprotein and obesity metabolism disorders in T2DM patients (3, 5). Oxidative stress occurs when the production of reactive oxygen species exceeds the capacity of antioxidant defense mechanisms and is produced by several drugs and pathological conditions, such as DM (8, 9). It has been shown to contribute to insulin resistance through activation of stress-sensitive signaling pathways, including glycation reactions (2, 4). 

Alpha-lipoic acid (ALA) is a naturally-occurring compound that has several bioactive functions, such as anti-oxidation influencing the glucose level regulation and recycling other antioxidants, such as glutathione and vitamins C and E (10–13) and reducing blood lipids (14–16). It has also been shown effective in enhancing the symptoms of peripheral diabetic polyneuropathy and diabetic nephropathy without serious adverse effects (13, 17–19). 

Recently, researchers tested its benefits for the treatment of traumatic brain injury, Alzheimer’s disease, and Parkinson’s disease and reported good results (12). 

A combination of intensive insulin treatment and ALA showed efficacy in normalizing hyperglycemia, relieving oxidative stress, and improving the beta-cell function and insulin sensitivity (1, 20–22). 

To the best of our knowledge, no systematic review evaluated the efficacy of ALA in DM patients. Accordingly, the present study aimed to investigate these outcomes in a meta-analysis framework.

## Experimental

We reported this systematic review and meta-analysis based on the Preferred Reporting Items for Systematic Review and Meta-analysis (PRISMA) statement and performed all steps according to the Cochrane Handbook (23). All steps of this study were prespecified, and the protocol was registered on PROSPERO (CRD42018083429).

*Literature Search Strategy*

We searched Medline (via PubMed), EBSCO, Web of Science, Scopus, and the Cochrane Library for relevant published studies up to May 20, 2017 (updated on April 21, 2018) with the following search terms: “Alpha-lipoic acid OR Thioctic Acid”, and “Diabetes Mellitus OR Metabolic Diseases”. 

*Study eligibility*

Studies satisfying the following criteria were included in our review:

Study design: studies that were described as randomized controlled trials with DM patients allocated to the treatment groups in a random manner.

Population: studies whose population was patients with type 1 and type 2 DM

Intervention: studies where the experimental group received the ALA (all doses are eligible either oral or intravenous administration).

Comparator: studies where the control group received a placebo.

Outcomes: studies reporting at least one of the following outcomes: glycated hemoglobin (HbA1c), fasting blood glucose (FBG), post-prandial blood glucose (PPBG), HDL, LDL, TG, TC, homeostatic model assessment (HOMA), glutathione peroxidase (GPx), and waist circumference.

We excluded studies in the following conditions: 1) animal experiments (not on human subjects), 2) studies that were not in the English language, and 3) reviews, case reports, case series, or studies whose full-text article was not available. 

*Study selection*

Eligibility screening was conducted in two steps, each by four independent reviewers (Ebada MA, Fayed N, Fayed L, and Alkanj S): a) title and abstract screening for matching the inclusion criteria, and b) full-text screening for eligibility to meta-analysis. Disagreements were resolved upon discussion. 

We selected prospective clinical trials assessing the efficacy of ALA in the management of DM. The bibliographies of the included studies and recent reviews were hand-searched.

*Data Extraction*

Four independent reviewers (Ebada MA, Fayed N, Fayed L, and Alkanj S) extracted the data from each included study using a pre-specified uniform data extraction sheet. 

The extracted data included the following domains: study ID, population, intervention, comparator, outcomes, study design, baseline characteristics of the study population, quality assessment domains, effect estimates of the study outcomes. In case of missing the mean or standard deviation (SD), we calculated them from the equations provided by Hozo *et al.* (24) or from those provided in the Cochrane Handbook (23).

*Assessment of risk of bias*

The quality of the retrieved RCTs was assessed according to the Cochrane Handbook for Systematic Reviews of Interventions 5.1.0 (updated March 2011) using the quality assessment table provided in the same book (part 2, Chapter 8.5). 

The Cochrane risk of bias assessment tool includes the following domains: sequence generation (selection bias), allocation sequence concealment (selection bias), blinding of participants and personnel (performance bias), blinding of outcome assessment (detection bias), incomplete outcome data (attrition bias), selective outcome reporting (reporting bias) and other potential sources of bias. The authors’ judgment is categorized as ‘Low risk,’ ‘High risk,’ or ‘Unclear risk’ of bias 

*Data Synthesis*

All data were pooled using RevMan version 5.3. For continuous outcomes, standardized mean differences (SMDs) and their 95% CIs were calculated. The Cochran Q test was used to compare the effect estimates among the included studies, and the inconsistency was assessed by testing the chi-square distribution of the Cochran Q values with *p* less than 0.1 indicating a significant statistical heterogeneity. A quantitative measure of heterogeneity across studies was also investigated using the I^2^ statistic where studies with I^2^ values of less than 40% were considered as having an acceptable level of statistical heterogeneity. In the case of significant heterogeneity, we used a random-effects model meta-analysis.

## Results

*Literature Search results*

**Figure 1:**
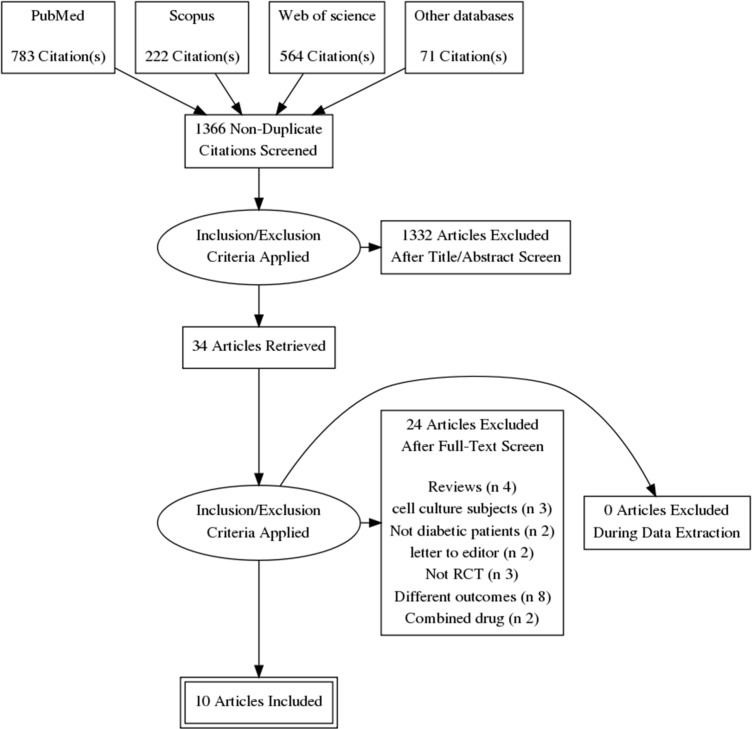
The flow diagram of the literature search and study selection

Our electronic literature search produced 1540 citations, which were abstracted to 1366 after duplicates′ removal using Endnote X8.0.1. An additional 1332 records were excluded during abstract screening, and the full-texts of 34 articles were examined in detail. Finally, we included 10 RCTs (1, 2, 4, 12, 13, 22 and 25–28) in quantitative synthesis (meta-analysis). The flow diagram of the literature search and study selection is shown in Figure 1.

*Demographics and Characteristics of the included studies′ population*

The included patients were T1DM in Hegazy *et al.*, both types in Ziegler *et al. *and uncomplicated T2DM in the remaining studies (Zhao *et al.* recruited DM patients who were suffering from acute cerebral infarction as well). The follow-up duration ranged from three weeks to six months. 

The daily doses of ALA were 300 and 600 mg orally or intravenously. A summary of the baseline characteristics of the study patients is shown in Table 1.

*Risk of bias*

**Figure 2:**
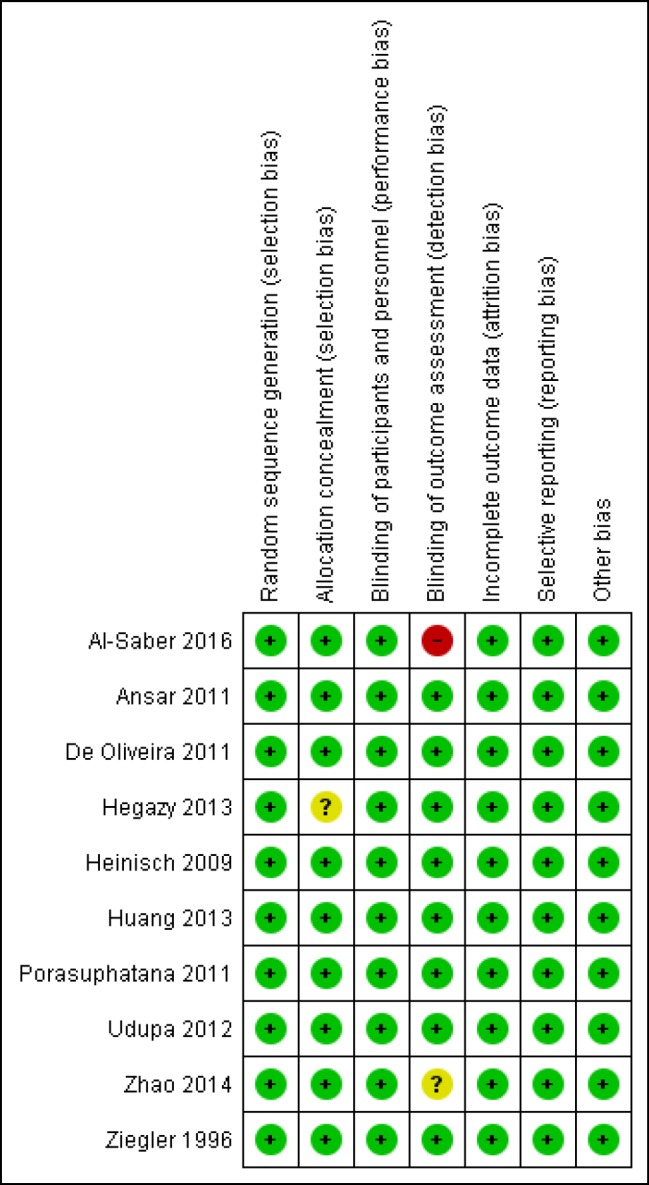
The risk of bias summary

All included studies were at low risk of bias in terms of random sequence generation, blinding of the participants, incomplete outcome data, and selective reporting. Nine out of 10 were at low risk of bias in terms of allocation concealment, and eight out of 10 were at low risk of blinding of the outcome assessment. The results were summarized with Review Manager version 5.3, and the graphical display is shown in Figure 2. Our evaluation showed that our systematic review and meta-analysis included high-quality studies.

*Efficacy outcomes of LEV*

*Glycated hemoglobin*

**Figure 3:**
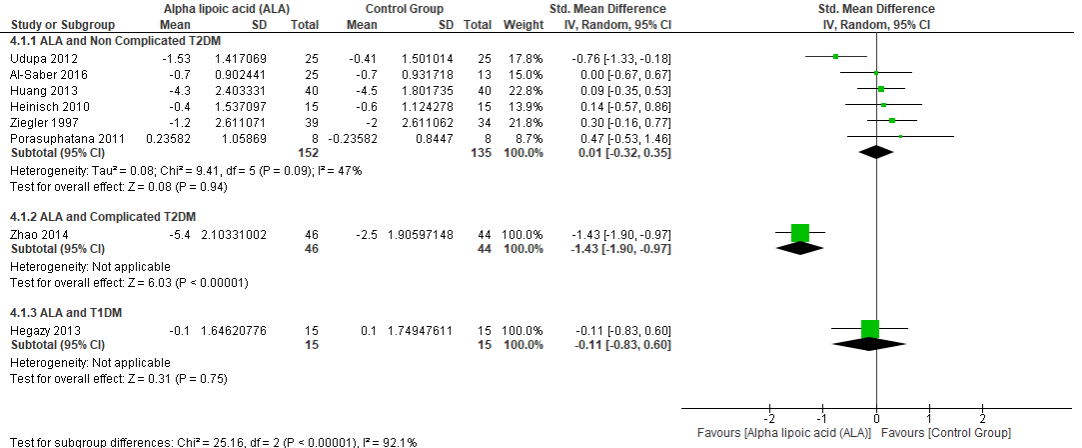
Forest plot of ALA *vs. *placebo, outcome: Glycated hemoglobin

Six studies (1, 4, 22 and 25–27) reported HbA1c in uncomplicated T2DM patients (n = 287 Participants), the overall SMD did not favor either of the two groups (SMD = 0.01, 95% CI [-0.32, 0.35]; *p* = 0.94) as shown in Figure 3.

In terms of T1DM, Hegazy *et al. * demonstrated that there was no significant difference between the two groups (SMD = -0.11, 95% CI [-0.83, 0.60]; *p* = 0.75) (13). 

On the other hand, one study by Zhao *et al. *reported HbA1c in complicated T2DM and ALA was superior to placebo (SMD = -1.43, 95% CI [-1.90, -0.97]; *p* < 0.00001) (12).

*Fasting blood glucose*

In terms of T1DM, Hegazy *et al*. showed no significant difference between the two groups (SMD = 0.00, 95% CI [-0.72, 0.72]; *p* = 1.00) (13). Moreover, one study investigated the effect of ALA on FBG in complicated T2DM (12). In this study, ALA was superior to placebo (SMD = -0.95, 95% CI [-1.39, -0.51]; *p *< 0.0001). 

**Figure 4:**
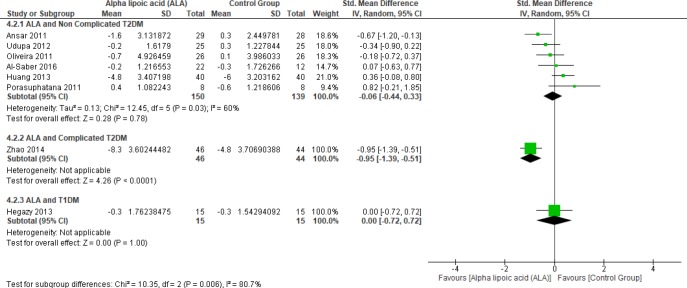
Forest plot of ALA *vs. *placebo, outcome: Fasting bl. Glucose

**Figure 5:**
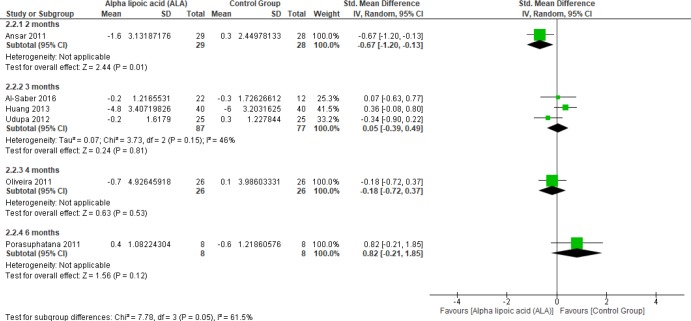
Forest plot of subgroup analysis of ALA-treated group according to Months, outcome: Fasting blood Glucose

Six studies (1, 2, 4, 22 and 27-28) reported the effect of ALA on FBG in uncomplicated T2DM patients (n = 289 Participants). The overall SMD did not favor either of the two groups (SMD = -0.06, 95% CI [-0.44, 0.33]; *p* = 0.78) as shown in Figure 4. The pooled studies were heterogeneous (*p *= 0.03; I² = 60%). Heterogeneity was resolved by subgroup analysis according to follow up periods (two, three, five, and six months) as shown in Figure 5. One study reported FBG in two months, ALA was superior to placebo (SMD = -0.67, 95% CI [-1.20, -0.13]; *p* = 0.01) (2). While three studies reported FBG in three months with no significant difference between the two groups (SMD = 0.05, 95% CI [-0.39, 0.49]; *p* = 0.81) (1, 22 and 27). The pooled studies were homogeneous (*p* = 0.15); I² = 46%). 

One study reported FBG in four-months follow-up period with no significant difference between the two groups (SMD = -0.18, 95% CI [-0.72, 0.37]; *p* = 0.53) (28). Only one study by Porasuphatana *et al*. reported FBG in six months with no significant difference between the two groups (SMD = 0.82, 95% CI [-0.21, 1.85]; *p* = 0.12).

*Post-prandial blood glucose*

**Figure 6:**
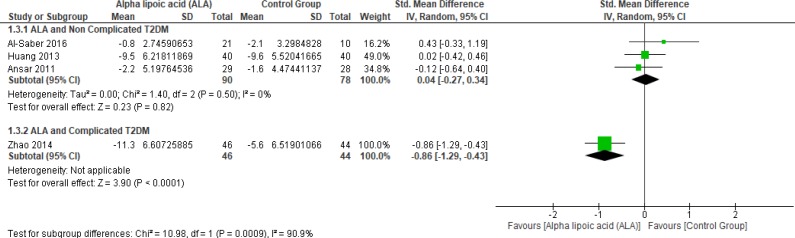
Forest plot of ALA *vs. *placebo, outcome: Post-prandial bl. Glucose

In terms of uncomplicated T2DM, three studies reported PPBG (n = 168 Participants) (2, 22 and 27). The overall SMD did not favor either of the two groups (SMD = 0.04, 95% CI [-0.27, 0.34]; *p* = 0.82) as shown in Figure 6. The pooled studies were homogeneous (*p* = 0.50; I² = 0%). 

While one study reported PPBG in complicated T2DM and ALA was superior to placebo (SMD = -0.86, 95% CI [-1.29, -0.43]; *p* < 0.0001) (12).

*Triglycerides*

**Figure 7:**
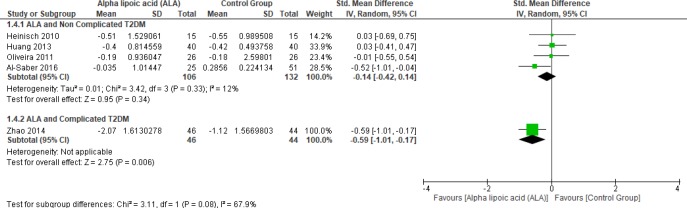
Forest plot of ALA *vs. *placebo, outcome: Triglycerides

In terms of uncomplicated T2DM, four studies reported TG (n = 238 Participants) (22, 25 and 27–28). The overall SMD did not favor either of the two groups (SMD = -0.14, 95% CI [-0.42, 0.14]; *p* = 0.34) as shown in Figure 7. The pooled studies were homogenous (*p* = 0.33); I² = 12%). On the other hand, only one study reported triglycerides in the patients with complicated T2DM; the ALA group was superior to the placebo group (SMD = -0.59, 95% CI [-1.01, -0.17]; *p* = 0.006) (12).

*Total Cholesterol*

In terms of complicated T2DM one study by Zhao *et al*. (2014) reported blood TC and ALA was superior to placebo (SMD = -1.43, 95% CI [-1.89, -0.96]; *p* < 0.00001) (12). 

**Figure 8:**
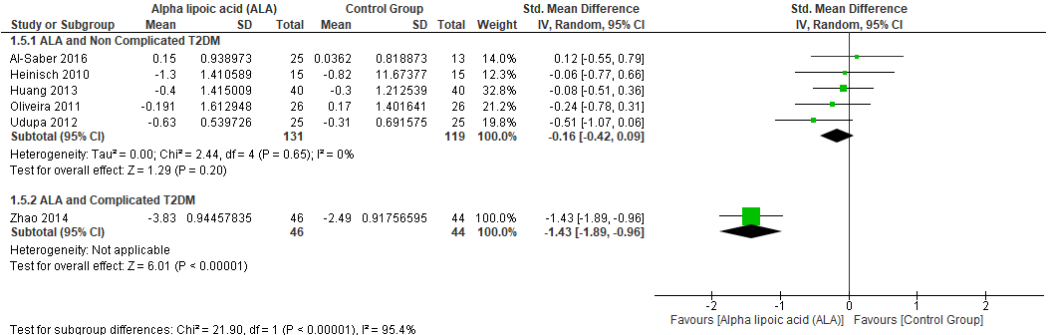
Forest plot of ALA *vs. *placebo, outcome: Total Cholesterol

On the other hand, five studies (1, 22, 25 and 27–28) reported in TC uncomplicated T2DM (n = 250 Participants). The overall SMD did not favor either of the two groups (SMD = -0.16, 95% CI [-0.42, 0.09]; *p* = 0.20) as shown in Figure 8. The pooled studies were homogenous (*p* = 0.65); I² = 0%).

*Low-density lipoprotein*

**Figure 9:**
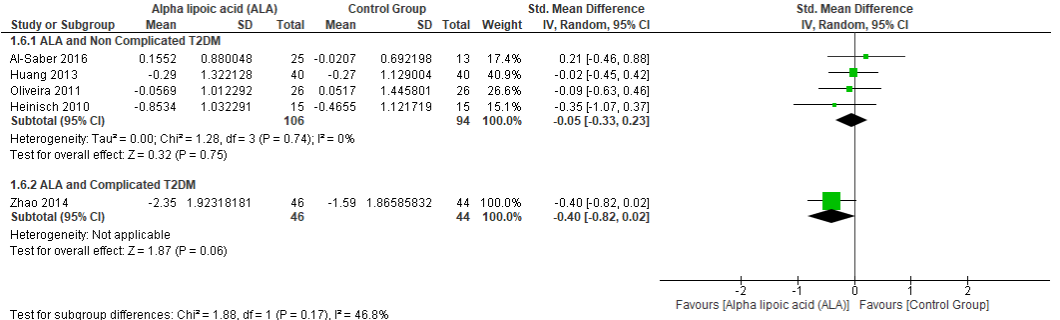
Forest plot of ALA *vs. *placebo, outcome: Low density lipoprotein

Four studies (22, 25 and 27–28) reported LDL in uncomplicated T2DM (n = 200 Participants). The overall SMD did not favor either of the two groups (SMD = -0.05, 95% CI [-0.33, 0.23]; *p* = 0.75) as shown in Figure 9. The pooled studies were homogenous (*p* = 0.74; I² = 0%), while one study by Zhao *et al*. (2014) reported LDL in complicated T2DM with no significant difference between the two groups (SMD = -0.40, 95% CI [-0.82, 0.02]; *p* = 0.06) (12).

*High-density lipoprotein*

**Figure 10:**
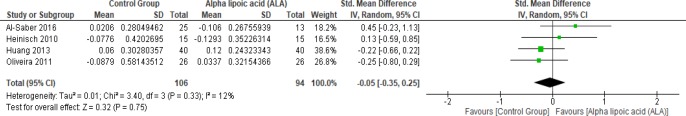
Forest plot of ALA *vs. *placebo, outcome: High Density Lipoprotein

Four studies (22, 25 and 27–28) reported HDL in uncomplicated T2DM (n = 200 Participants). The overall SMD did not favor either of the two groups (SMD = -0.05, 95% CI [-0.35, 0.25]; *p* = 0.75) as shown in Figure 10. The pooled studies were homogenous (*p* = 0.33; I² = 12%).

*Waist circumference*

**Figure 11:**
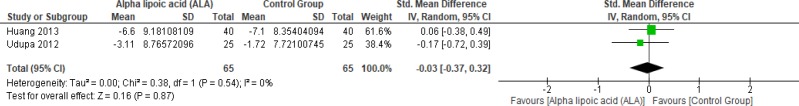
Forest plot of ALA *vs. *placebo, outcome: Waist circumference

Two studies reported waist circumference (n = 130 Participants) (1, 22). The overall SMD did not favor either of the two groups (SMD = -0.03, 95% CI [-0.37, 0.32]; *p* = 0.87) as shown in Figure 11. The pooled studies were homogenous (*p* = 0.54); I² = 0%).

*Glutathione peroxidase*

**Figure 12:**
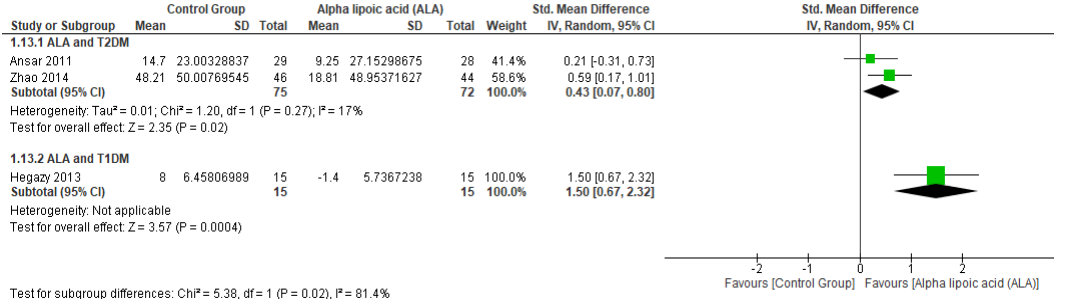
Forest plot of ALA *vs. *placebo, outcome: Glutathione peroxidase

Two studies (2, 12) reported Gpx (n = 147 Participants). The overall SMD favored ALA-treated group over placebo group in T2DM (SMD = 0.43, 95% CI [0.07, 0.8]; *p* = 0.02) as shown in Figure 12. The pooled studies were homogenous (*p* = 0.27); I² = 17%), in T1DM (SMD = 1.50, 95% CI [0.67, 2.32]; *p* = 0.0004).

*Homeostatic model assessment insulin resistance*

**Figure 13:**
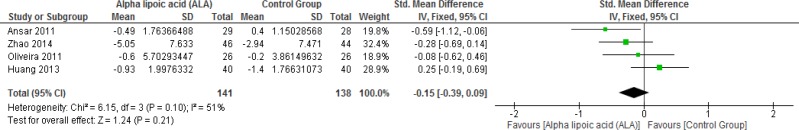
Forest plot of ALA *vs. *placebo, outcome: Homeostatic model assessment insulin resistance

Four studies reported HOMA-IR in T2DM (n = 279 Participants). The overall SMD did not favor either of the two groups in this outcome (SMD = -0.15, 95% CI [-0.39, 0.09]; *p* = 0.21) as shown in Figure 13. The pooled studies were homogenous (*p *= 0.01; I² = 51%).

## Discussion

Alpha-lipoic acid is present in low quantities in foods and is used as a pharmaceutical and dietary agent. It also activates the electron transport chain in mitochondria and further induces heme oxygenase-1, reducing Reactive oxygen species release and thus it has been studied globally in diabetic patients (27, 30–32). Diabetic patients are known to have low glutathione levels, and ALA increased glutathione levels through increasing cellular uptake of the cysteine required for its synthesis

*Summary of main results*

Our meta-analysis of 10 RCTs provides level 1 evidence that ALA does not improve the metabolic abnormalities of uncomplicated T2DM. HbA1c, TG, TC levels in the blood, waist circumference, and HOMA index did not differ significantly between ALA and placebo groups in uncomplicated T2DM. 

Evidence was not sufficient in terms of the complicated T2DM and T1DM. In the study by Zhao *et al*., ALA was superior to placebo in patients with complicated T2DM (12). In the study of Hegazy *et al*., ALA was superior to placebo in terms of GPx production in children and adolescents with T1DM (13).

*Efficacy with long-term use*

The follow-up period varied between the included studies: three weeks (in Zhao *et al.* and Heinisch *et al.* two months in Ansar *et al.*), three months (in Udupa *et al.,* Huang *et al.* and Al-Saber *et al.*), four months (in Ziegler *et al.,* Hegazy *et al.* and Oliveira *et al.*), and six months (in Porasuphatana 2011). In terms of FBG, ALA was superior in the study of 2-month follow up period and failed in the studies of longer duration, while ALA showed no statistical difference between both groups in other outcomes.

*Agreement and disagreement with previous studies*

To our knowledge, no systematic review and meta-analysis investigated the efficacy of ALA for the management of DM. In our meta-analysis, we included 10 RCTs (1, 2, 4, 12, 13, 22 and 25–28) demonstrating the difference between ALA-treated group and placebo group in glycemic status as well as different parameters and suggested using it as add-on therapy, and it might be effective against oxidative stress in DM patients. 

On the other hand, two studies (REF) reported that ALA might not have an additive effect as an antioxidant in terms of reduction of insulin resistance or improvement of lipid profile. While the remaining three studies: Hegazy *et al*. showed that ALA might have a role in preventing the development of diabetic cardiomyopathy in T1DM, Heinisch *et al*. showed that ALA treatment improves endothelium-dependent vasodilatation in the patients with T2DM, and Ziegler *et al*. showed that ALA might slightly improve Cardiac autonomic neuropathy in NIDDM patients.

*Strength points and Limitations*

Our meta-analysis has several strength points: 1) We determined Search methods and performed comprehensive search using many electronic databases; 2) in our systematic review we followed PRISMA checklist when reporting this manuscript; 3) All steps were done in strict correspondence with Cochrane handbook of systematic reviews for interventions, and 4) we performed subgroup analysis according to DM type and follow up period.

However, there are a few limitations in our meta-analysis. On comparing the individual studies results, it is clear that data on complicated T2DM and T1DM were scarce. Only one study was found for each of the complicated T2DM and T1DM. Interestingly, the study by Zhao *et al*. which enrolled patients with complicated T2DM showed the most favorable effects of ALA. Based on this, we recommend further studies with a larger sample size to investigate different doses of ALA in complicated T2DM and T1DM patients.

*The overall completeness and quality of Evidence*

The quality of this evidence is reasonable as it is based on studies of high quality as indicated by the risk of bias assessment (ROB). Additionally, the discontinuation rate was 48 patients out of the total of 553 patients in the 10 randomized controlled studies. These discontinuations, which are less than 10%, were balanced between the two groups.

**Table 1:** The characteristics of the included studies (n = 553 patients)

**Study ID**	**Country**	**Type of Diabetes**	**Number of Patients**	**Mean age (Years)**		**Follow up (months)**	**Gender**		**Dropout**	**ALA dose** **and Route of administration**	**Findings**
			**ALA/Control**	**ALA**	**Control**		**ALA M/F**	**Control** **M/F**	**ALA/Control**		
Hegazy 2013	Egypt	Diabetes type 1	15/15	11.1 ± 2.3	11.9 ± 1.4	4	7/8	7/8	No dropouts	Oral administration of (300 mg) ALA twice daily	ALA may have a role in preventing the development of diabetic cardiomyopathy in type 1 diabetes.
Huang 2013	China	Diabetes type 2	40/40	49.6 ± 10.5	50.8 ± 9.7	3	15/25	14/26	10/11	Intravenous infusion of (600 mg) ALA once daily	ALA might not have an additive effect
Udupa 2012	India	Diabetes type 2	25/25	53.5 ± 1.4	53.8 ± 2.1	3	12/13	15/10	10	Oral administration of (300 mg) ALA	ALA showed an improvement in insulin sensitivity. Since it differs in improving different parameters and can be used as add-on therapy in patients with type 2 diabetes mellitus
Zhao 2014	China	Diabetes type 2	46/44	71.6 (60-92)		1	49/41		No dropouts	Intravenous Injection of 600 mg ALA in 250 mL 0.9% sodium chloride	ALA was safe and effective in the treatment of aged T2DM complicate with acute cerebral infarction, significantly reducing the patient's oxidative stress, blood glucose, and lipid levels and being able to improve islet function.
Al-Saber 2016	Kingdom of Bahrain	Diabetes type 2	35/18	52.4 (6.51)	51.9 (5.07)	3	33/2	13/5	No dropouts	Oral administration of 1OO, 150 and 200 mg of ALA at two divided doses	ALA may Offer benefits in the diabetic population.
Ansar 2011	Iran	Diabetes type 2	29/28	49 ± 9.07	51.82 ± 8.25	2	6/23	8/20	No dropouts	Oral administration of 300 mg ALA daily	This study supports the use of ALA as an antioxidant in the care of diabetic patients.
De Oliveira 2011	Brazil	Diabetes type 2	26/26	3: 39-49 9: 50-59 11: 60-69 3: > or =70	2: 39-49 6: 50-59 13: 60-69 5: > or =70	4	16/10	15/11	No dropouts	Oral administration of 600 mg ALA daily	No beneficial effect of treatment with this antioxidant in terms of reduction of insulin resistance or improvement of lipid profile.
Heinisch 2009	Austria	Diabetes type 2	15/15	55 ± 8	56 ± 6	1	NA		No dropouts	Intravenous Injection of 600 mg ALA daily	ALA treatment improves endothelium-dependent vasodilatation in patients with type 2 diabetes.
Porasuphatana 2011	Thailand	Diabetes type 2	30/8	45.7 ± 1.68	58.6 ± 6.7	6	9/21	1/7	No dropouts	Oral administration of 300, 600, 900 and 1200 mg\day of ALA	Oral ALA treatment could improve the glycemic status and is slightly effective against oxidative stress in patients with type 2 DM with tolerable minor adverse events.
Ziegler 1996	Germany	Diabetes type 2	39/34	57.9 ± 6.6		4	24/15	24/10	17	Oral administration of 800 mg ALA daily	Treatment with ALA using an oral dose of 800 mg/day for four months may slightly improve NIDDM patients

LA = Alpha lipoic acid; M/F = Male/Female; continuous variables are presented as mean (SD).

## Conclusion

In conclusion, our analysis showed no significant difference between ALA and placebo in terms of HBA1C, LDL, HDL, TC, and TG reduction in uncomplicated T2DM. However, in terms of GPx, ALA was significantly superior to placebo in T1DM and T2DM. Further studies should employ larger sample sizes and test different doses of ALA in DM patients.
